# Human papillomavirus vaccine uptake and associated factors among adolescent girls in high schools of Nekemte city, Western Ethiopia, 2020

**DOI:** 10.1186/s12905-023-02702-8

**Published:** 2023-10-28

**Authors:** Genet Hailu, Desalegn Wirtu, Tariku Tesfaye, Motuma Getachew

**Affiliations:** Department of Public Health, Wollega University Institute of Health Science, P.O. Box 385, Nekemte, Ethiopia

**Keywords:** HPV, Uptake, Vaccine, Nekemte, Western Ethiopia

## Abstract

**Background:**

Cervical cancer is the leading cause of cancer death in adult women in the developing world including Ethiopia. To combat cervical cancer, the World Health Organization (WHO) recommends that girls aged 9–14 years have to take the human papillomavirus vaccine. However, there is a lack of information regarding the uptake of human papillomavirus vaccine in the study area. Therefore, this study aimed to assess the Human Papilloma Virus vaccine uptake and associated factors among adolescent girls in high schools of Nekemte City, Western Ethiopia, 2020.

**Methods:**

A cross-sectional study design was employed among adolescent girls attending grade 9 and age 15 enrolled at schools in Nekemte City from July 15–30, 2020. Six hundred twenty-six (626) randomly selected adolescent girls were interviewed. The data were entered into Epi Info 7 and analyzed by SPSS 25. Multivariable analysis was computed and a *P*-value < 0.05 was taken as a cut-off point to declare the statistically significant association.

**Result:**

The uptake of the HPV vaccine was 61.2%, 95%CI (57.2%, 65%). The Place where adolescents grow up (AOR = 3.46, 95%CI [1.95,6.15]), having a mobile phone(AOR = 1.71, 95%CI [1.05, 2.79]), ever heard about HPV (AOR = 5.69, 95%CI [1.33, 24.27]), ever heard about HPV vaccine(AOR = 1.917, 95%CI [1.002, 3.667]), Ever had sexual intercourse (AOR = 3.04, 95% [1.49,6.20]) and Perceived risk of towards HPV(AOR = 4.63 [2.49, 8.63]) has shown statistically significant association with Uptake of the HPV vaccine.

**Conclusion:**

Nearly two-thirds of the study participants had taken at least one dose of the HPV vaccine. It is better if health information on HPV is disseminated considering the available technology like mobile phones and reaching rural girls.

## Introduction

Human Papillomavirus (HPV) is the most common type of viral infection responsible for a wide range of disorders including precancerous lesions that will advance to cancer. There are more than 150 types of HPV [1]. More than 70% global burden of cervical cancer is attributed to the two commonest types of HPV 16 &18 [2].

HPV persistence is one of the most important factors for predicting the risk of recurrence. The risk of Cervical intraepithelial (CIN2 +) recurrence increased with increased HPV persistence for up to 1 year [3].

Cervical cancer is the leading cause of cancer death among adult women in the developing world and the second most common cancer among women worldwide [4]. Cervical cancers comprise 84% of all HPV-related cancers worldwide [5] and the burden is projected to increase to almost 460,000 deaths by 2040 [6].

In 2018, the estimated incidence of cervical cancer was 570,000 with approximately 311,000 women dying from cervical cancer & with more than 85% of these deaths occurring in low- and middle-income countries [5–8]. In Ethiopia, the estimated number of cervical cancer incidences & deaths in 2018 is 6294 & 4884. In the country cervical cancer ranks as the 2^nd^ leading cause of female cancer in women aged 15 to 44 years [9].

The chance of being infected by genital HPV increases when the women are at a younger age, initiate sexual intercourse early, have multiple sexual partners, or have a partner who has multiple partners or an HPV infection and does not use a condom [10, 11]. There is no cure for the HPV disease; however, persistent HPV infection can be prevented by completing the HPV vaccine series before the onset of sexual activity [12, 13].

HPV vaccination is one of the strategies of primary prevention of cervical Cancer. It reduces the development of precancerous lesions and significantly contributes to deflecting the incidence of cervical cancer. The HPV vaccine is the most cost-effective public health measure against cervical cancer. Because of this, it can be considered a cornerstone for the prevention of invasive cervical cancer [14].

World Health Organization (WHO) recommends that the primary target group for the HPV vaccine is girls aged 9–14 years [15]. The early vaccine provides a superior immune response, and it has been shown that 2 doses in 9-to 13-year-old children elicited even higher antibody levels than 3 doses in young adults [16]. In countries where at least 50% of eligible females were vaccinated, HPV 16, and 18 infections decreased by almost 70% [17].

In 2017, WHO identified cervical cancer as a public health priority and recommended that all countries have to include HPV vaccines within their national immunization programs. By 2017, globally 71 countries (37%) had introduced the vaccine into their national immunization programs for girls, and 11 countries (6%) also for boys [18].

The uptake of the HPV vaccine remains sub-optimal worldwide, which threatens to undermine its individual and public health impact [19]. Scholars reveal that the uptake of HPV vaccine varies globally ranging from 31% in Kenya to 81% in Malaysia among adolescents & young girls [20–28]. Besides, in recent years, HPV vaccination has suffered from growing public distrust and criticism in Europe [29]. A study done in Denmark on the uptake of the HPV vaccine shows a declining pattern for girls born after 2000 [30].

Studies dictate that the factors associated with HPV vaccine uptake were: Age [22, 31], race, income, parents’ education, knowledge about cervical cancer, awareness about HPV Vaccine, knowing the place of Vaccine center [22] parental & health provider influence[23, 26, 28, 31, 32] Having sexual intercourse, religious/spiritual belief [33], Diverse HPV vaccine outlet, receiving vaccine full information [29], a concern of side effects [24, 30].

In Ethiopia, the vaccination is not incorporated in the national immunization programs yet, however, since 2015 it was intended to be provided for the primary target group recommended by WHO at school, However, due to the shortage of the vaccination only girls age 14 are currently receiving the vaccine. In December 2018, Ethiopia’s first nationwide immunization campaign against HPV reached over 2.4 million girls [34]. There is a lack of information regarding the uptake of HPV vaccination in the study area. Therefore, the objective of this study was to assess the Human Papilloma Virus Vaccine uptake and associated factors among adolescent girls in High schools of Nekemte City, Western Ethiopia,2020.

## Method and materials

### Study area and period

This study was conducted in Nekemte city, the capital of East Wollega Zone, which is located at a distance of 314 km to the West of the capital city, Addis Ababa, in Oromia National Regional State. According to the 2011 E.C. estimated by CSA to be a total of 117,771 populations of which 60,100 males and 57,671 females. There are about 30,019 youths ages between 15 – 29 years of which 15,069 are males and 14,950 females. In the city, there is one Government University and 2 private university colleges, one Teacher training college (TTC), 4 preparatory schools (grade 11 and 12), 12 high schools,2 referral Hospitals, and 2 health centers that are giving services to the community. The study was conducted from July 15–30, 2020.

### Study design

A cross-sectional study design was conducted among adolescent girls aged 15 years attending high schools in Nekemte City.

## Population

### Source population

All adolescent girls attending grade 9 and age 15 enrolled in the high schools of Nekemte City.

### Study population

Randomly selected adolescent girls attending grade 9 and age 15 enrolled in the high schools in Nekemte City during the study period.

#### Inclusion criteria

Adolescent girls whose age was 15 years & enrolled in grade 9 at high schools in Nekemte city were included. This age group was selected because the Vaccine was given in the previous year which is for grade 8 students whose age is 14.

#### Exclusion criteria

Adolescent girls who were unable to respond to a questionnaire due to illness were excluded. Adolescents who were unable to respond due to medical illness were excluded from the study.

### Sample size determination

The sample size needed for the study will be calculated using the single population proportion formula with the assumption of a 95% confidence level, 5% margin of error tolerated, assuming *P*= 50% (Because there are no studies conducted in Ethiopia or low-income countries on a single age group of adolescents).

$$\mathrm{n}=\frac{{\left({\mathbf{Z}}_{{\varvec{\upalpha}}}/2\right)}^{2}\mathbf{p}\left(1-\mathbf{p}\right)}{{\mathbf{d}}^{2}} ,\mathrm{ n}=\frac{{\left(1.96\right)}^{2}*\left(0.5\right)*\left(1-0.5\right)}{{\left(0.05\right)}^{2}}=384$$

$$\mathrm{n}=384+\mathrm{non}-\mathrm{response rate }\left(10\%\right)=307+31=423$$

where: n - Minimum sample size, $Z_\frac\alpha2$ critical value at 95% Confidence interval

P – Proportion of adolescent girls with the HPV vaccine uptake =0.5

d – Margin of error 5%

Then, multiply it by 1.5 (design effect) it is = 423*1.5 = 634.5 ~ **635**

#### Sampling technique

The data collection period overlaps with the COVID-19 outbreak and because of this, the schools were closed. Therefore, the ethical clearance was secured from the respective body, and the adolescent girls were reached at their residences. Firstly, four of the six sub-cities in Nekemte City named “Derge”, “Cheleki”, “Burka Jato”, and “Bekenisa kese” were selected. Then, from each sub-city, four zones were randomly selected and all adolescent girls enrolled in grade 9 in the zones were registered. Then the total sample size was proportionally allocated to the selected sub-cities based on their respective number of adolescent girls. Moreover, the adolescent girls who participated in the study were selected randomly using computer-generated random numbers after ordering their first name combined with a household number.

#### Data collection tool and procedures

A Pre-tested structured questionnaire that incorporates the socio-demographic, HPV-related questions was developed from different kinds of literature and administered through face-to-face interviews. The structured questionnaire was first prepared in English then translated into Afan Oromo and retranslated back to English to check its consistency. Thirteen College and university female graduates participated in the study as data collectors and Two Master of Public health holders were supervising the data collection procedure. The training was given to the data collectors and supervisors; the training was given by dividing them into three groups to ensure the COVID-19 restriction precautions.

The recommended Personal Protective Equipment (PPE) like wearing masks, hand hygiene, and sitting at least 2 m apart were ensured during each training session and in-between contacts. They were also, strictly advised and supervised to follow the recommended PPE measures during data collection. Moreover, the collected data were checked daily by the supervisors and investigator for any inconsistencies and clarifications. A Pretest was done on 5% of the size of study participants in the sub-cities that were not included in the study two weeks ahead of the actual data collection.

#### Data processing & analysis

The collected data were entered into Epi info version 7 statistical software and cleaned and analyzed by using SPSS version 25. The findings of this study were presented in the form of tables, figures, and narration. Mean, Median and standard deviation were used to summarize the continuous variables. Bivariable and multivariable analyses were performed to ascertain factors associated with the uptake of the vaccine. Those variables with a P value of < 0.25 in the bivariable analysis was considered a candidate for a multi-variable logistic regression model. Adjusted odds ratios with their 95% confidence intervals were computed to measure the strength of associations, and statistical significance was declared at *P* < 0.05.

## Result

### Socio-demographic characteristics

A total of 626 adolescent girls enrolled in grade 9 and aged 15 years attending the high schools of Nekemte City participated in the study making a response rate of 98.58%. Nearly three fourth 457(73%) of the participants were attending public school. Four hundred ninety-two (78.6%) of the girls were grown up in urban areas. The majority of the respondents belonged to the Oromo ethnic group and followed the protestant religion 299(47.8%).

Three-fourths of the respondents 469(74.9%) live with their mother and father. Most of the study participants 443(70.8%) reported their perceived family status as middle income. Four hundred twenty (67.1%) have owned a mobile phone and three hundred sixty-eight (59.8%) had at least one social media account Table 1.

**Table 1:** Socio-demographic characteristics of adolescent girls in Nekemte city high schools Western, Ethiopia, 2020

Characteristics		Frequency (N = 626),	Percentage (%)
School type	Public	457	73
	Private/NGO	169	27
Grow up place	Urban	492	78.6
	Rural	134	21.4
Ethnicity	Oromo	542	86.6
	Amhara	60	9.6
	0thers	24	3.8
Religion	Protestant	299	47.8
	Orthodox	224	35.8
	Muslim	75	12.0
	Others	**28**	4.4
Currently Living with	Mother and father	469	74.9
	Father only	25	4.0
	Mother only	48	7.7
	Brother/sister	35	5.6
	Relatives	49	7.8
Educational status of Father	Didn’t attend formal Education	89	14.2
	Primary school (1–8)	125	20.0
	High school (9–12)	149	23.8
	College and above	263	42.0
Occupational status of Father	Govt employee	211	33.7
	Farmer	106	16.9
	Merchant	154	24.6
	Daily laborer	97	15.5
	NGO employee	49	7.8
	Other	9	1.4
Educational status of Mother	Didn’t attend formal Education	93	14.9
	Primary school (1–8)	192	30.7
	High school (9–12)	127	20.3
	College and above	192	30.7
Occupational status of Mother	House wife	262	41.9
	Govt employee	137	21.9
	Merchant	170	27.2
	NGO employee	19	3.0
	Daily laborer	38	6.1
	Don’t know	1	0.2
Perceived family status	Rich	66	10.5
	Very rich	8	1.3
	Middle income	443	70.8
	Poor	98	15.7
	Very poor	11	1.8
Having a Mobile phone	Yes	420	67.1
	No	206	32.9
Social media account	Yes	368	59.8
	No	258	41.2

### Awareness of Human Papillomavirus and vaccine

**Fig. 1:**
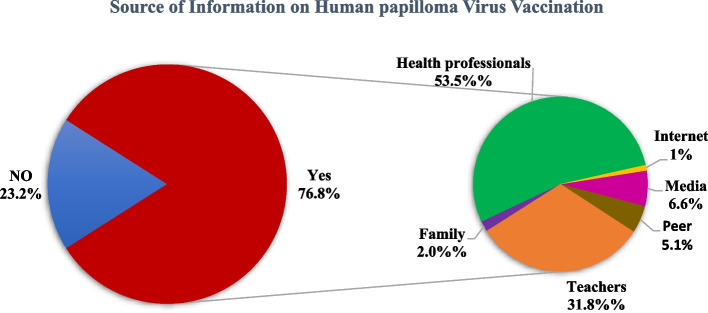
Source of information for Hman Papillomavirus vaccine among adolescent girls in Nekemte city high schools Western, Ethiopia, 2020

More than three-fourths of 486(77.6%) of the study participants have ever heard about human papillomavirus. The two most common sources of information on the Human Papillomavirus were Health professionals 258(52.8%) and teachers 179(36.6%) [Fig. 1]. Eighty-four (17.5%) respondents replied that they knew the target group of the HPV vaccine and all of them preferred to mention “adolescent girls” rather than specifying the age group. A quarter of the respondents, 126 (25.9%) had perceived that they were at risk of human Papilloma virus.

### History of sexual intercourse

Ninety-one (14.5%) of the respondents had ever engaged in sexual intercourse and among them, 85(93.4%) did engage in the last year. The median age at first sexual intercourse was 14(SD ± 0.64). In their recent sexual history, most of the respondents 63(73.3%) had mentioned that their sexual partners were boyfriends whereas 7(8.1%) and 4(4.7%) were causal partners and teachers respectively.

### Uptake of Human Papillomavirus

**Fig. 2:**
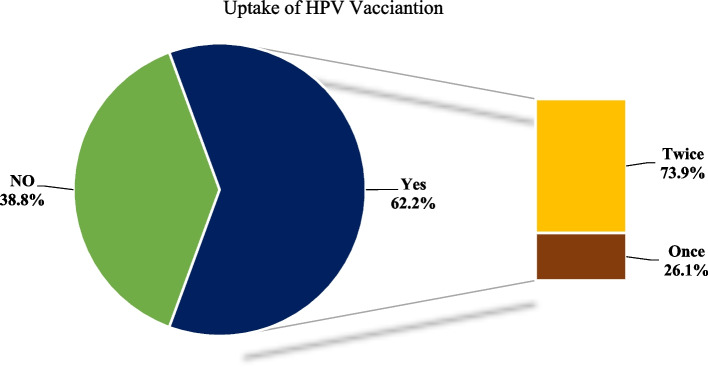
Uptake of Human Papillomavirus vaccine among adolescent girls in Nekemte city high schools Western, Ethiopia, 2020

Of the total respondents, nearly two-thirds of 383(61.2%) had taken the HPV vaccine last year (when they were in grade eight and age 14). Among those who took the HPV vaccine, 283(73.9%) had taken the full dose (two) [Fig. 2]. The top motivator for HPV vaccine was School health education 281(73.4%) followed by Health professionals’ advice 46(12%) while peer influence, Media advertising, and family support accounted for 27(7%), 24(6.3%), and 5(13%) respectively. The most common reasons mentioned for missing the second dose by those who took only one dose were *not trusting the vaccine* 45(45%) followed by *fear of becoming infertile* 17(17%) while 8(8%) of them were *absent from school* on the day of delivery of the second dose.

### Reason for not taking the vaccination

**Fig. 3:**
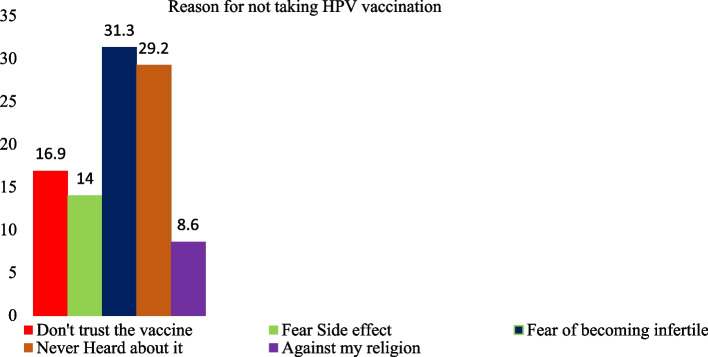
Reasons for not taking the human Papillomavirus vaccine among adolescent girls in Nekemte city high schools Western, Ethiopia, 2020

On the other hand, among those who didn’t take the HPV vaccine, fear of becoming infertile 76 (31.3%) and not ever knowing the vaccine 71(29.2%) were mentioned as the major reasons for not taking the vaccine. Besides, forty-one (16.1%) of the respondents expressed mistrust regarding the vaccine, and thirty-four (14%) feared side effects as an excuse to escape the vaccine [Fig. 3].

Moreover, almost three-fourths, 448(71.6%) of the study participants believed that they decided by themselves to take the vaccine while a quarter, 153(24.4%) of them believed that the decision would be of their families.

### Factors associated with the uptake HPV vaccine

In multivariable analysis, after adjusting for the confounding variables place where the adolescent has grown up (AOR = 3.46, 95%CI [1.95,6.15]), Having mobile (AOR = 1.71, 95%CI [1.05, 2.79]), Ever heard about human papillomavirus (AOR = 5.69, 95%CI [1.33, 24.27], P = 0.019), Ever heard about the HPV vaccine (AOR = 1.917, 95%CI [1.002, 3.667],

Ever engaging in sexual intercourse (AOR = 3.04, 95% [1.49,6.20]) and perceived risk towards HPV (AOR = 4.63 [2.49, 8.63]) Table 2.

**Table 2:** Multi-variable analysis of factors associated with the uptake of HPV vaccine among adolescent girls in Nekemte city high schools in Western Oromia, Ethiopia,2020 (*N* = 626)

Characteristics		Uptake of HPV		COR (95%CI)	AOR (95% CI)
		Yes	No		
Place of growing up	Urban	335	157	3.82 [2.56, 5.71]	3.46 [1.95,6.15] **
	Rural	48	86	1	1
Father educational status	No formal education	44	45	1	1
	Primary & High school	170	104	1.67 [1.03, 2.71]	1.20 [0.61, 2.37]]
	College and above	169	94	1.84 [1.13, 2.99]	1.98 [0.927, 4.215]
Father Occupation	Not Employed	212	154	1	1
	Employed	171	89	1.39 [1.01, 1.94]	1.01 [ 0.56, 1.83]
Mother Education	No formal education	52	41	1	1
	Primary & High school	129	190	1.87 [1.17, 2.98]	1.20 [0.61,2.37]
	College and above	62	152	3.11 [1.88,5.15]	1.98 [0.98, 4.22]
Mother Occupation	Not Employed	276	194	1	1
	Employed	107	49	1.54 [1.05, 2.25]	1.14 [0.453, 2.74]
Perceived Family status	Poor	54	55	1	1
	Middle income	269	174	1.56 [1.03, 2.40]	.585 [.299, 1.4]
	Rich	60	14	4.37 [2.18, 8.72]	1.10[0.43, 2.79]
Living arrangement	Both parents	313	156	2.68 [1.67,4.29]	1.36 [0.68, 2.73]
	Single parent	34	39	1.16 [0.62, 2.19]	0.66 [0.28, 1.58]
	Sibling/Relative	36	48	1	1
Having a Mobile phone	Yes	280	140	2.00 [.42, 2.81]	1.71 [1.05, 2.79] *
	No	103	103	1	1
Ever Heard about HPV	Yes	324	162	2.75 [1.87, 4.04]	5.69 [1.33, 24.27] *
	No	59	81	1	1
Ever had sexual intercourse	Yes	66	25	1.82 [1.11, 2.97]	3.04 [1.49,6.20] *
	No	317	218	1	1
Perceived risk of acquiring HPV	Yes	110	16	5.14 [2.92,9.04]	4.63 [2.49, 8.63] **
	NO	206	154	1	1
Heard about the HPV vaccine	Yes	327	154	3.38 [2.27,4.96]	1.917 [1.002, 3.667] *
	No	96	76	1	1
Perceived decisive	My self	297	151	3.497 [1.510, 8.09]	1.64[0.495, 5.418]
person	My family	77	76	1.80 [0.75, 4.33]	0.696 [0.19, 2.43]
	Teacher/peers	9	16	1	1

^**^ = *P* < 0.01, * = *P* < 0.05  Statistically significant association

## Discussion

This study assessed the level of uptake of human papillomavirus and its associated factors like socio-demographic factors, and awareness of HPV and HPV vaccines.

In this study, the proportion of study participants who ever heard about Human papillomavirus was 77.6, 95%CI [73.3, 80.1]. This finding was higher than A systematic review done in the Middle East and Northern African region where 20% of adolescents reported that they knew about the human papillomavirus [35] and similarly, higher than a study conducted in Cameroon where 20.7% of adolescents in school girls were aware of the existence of human papillomavirus [36]. The difference would be attributed to the time difference between the studies and only specific age groups of adolescents were included in the recent study.

On the other hand, regarding the awareness of the HPV vaccine, this study dictated that 76.8%, 95%CI [73.1,80.1] of the adolescent girls were aware of the existence of the HPV vaccine. This finding was higher than the study conducted in Los Angeles County among Ethnic Minority girls about 61% had heard of about HPV vaccine. However, it is lower than studies conducted in Valencia, Spain [37], and Cameroon [36] where 97.4% and 87.1% of adolescent girls had ever heard about the HPV vaccine respectively. The difference might be due to the different age groups of adolescent girls who participated in the study, the time difference between the studies, and the study setting. Furthermore, the finding was nearly comparable with the study conducted on school girls in Melaka, Malaysia 77.6% had never heard about the HPV vaccine [22].

In this study, the uptake of HPV vaccine among adolescent girls was 61.2% 95%CI(57.2, 65%) which is higher than the study conducted in the United States of America, Ohio Country among adolescents aged 11–18 years 21.3% [28], Bali, Indonesia among adolescent girls (50.7%) [38], Uganda Lira district among female adolescents (50.4%) [29]. Besides, it is lower than the study conducted in Melaka Malaysia among school girls (77.9%) [22]. The gap might be attributed to the difference in the study setting, the age group of the study population included in the study, and the service outlet of the vaccine. However, the finding was nearly similar to a study conducted in Germany among 10^th^-grade students (60.2%) [27]. This might be due to both of the studies focusing on similar single-age and single-grade students.

In this study, among those who took the vaccine, the completion of the full dose (two doses) accounts for 283(73.9%). Similarly, a longitudinal study conducted in Kenya Eldoret shows that 72% of girls aged 9–13 have completed the recommended dose [3 doses] [39]. However, the finding in this study was higher than the study conducted in the Lira district of Uganda where the full dose uptake was 17.6% [31]. This might be due to the difference in the point of vaccine delivery as the later study includes places other than school and the time lapse between the two studies.

In this study, among those who didn’t take the vaccine, the common reason mentioned was Myth about the vaccine (31.3%) and never hearing about the vaccine (29.2%). Also, in a longitudinal study conducted in the Netherlands, fear of serious side effects (23.2%), and too little information about the vaccine availability (17.2%) [40]. In Ibadan Nigeria, the most common reason mentioned for not taking the vaccine was not knowing where to go for the HPV vaccine [85.8%] [41]. These studies dictate a common concern of the lack of information as a reason for not taking the vaccine.

In this study, having awareness about the HPV virus and vaccine were predictors of HPV vaccine uptake. Adolescent girls who had awareness about Human papillomavirus were 5 times more likely to take the HPV vaccination whereas those who had awareness of the HPV vaccine had 2 times more likely to take the HPV vaccine than those who didn’t have the awareness. Similarly, In Melaka Malaysia knowledge about cervical cancer, and hearing about the HPV vaccine were predictors of HPV vaccine uptake [22]. Also, a study done in the Lira district in Uganda having information on the Human papillomavirus showed a statistically significant association with the uptake of the HPV vaccine [31].

In a recent study, the residence has shown a statistically significant association with HPV uptake as those who grew up in urban areas were 3.5 times more likely to receive the vaccine compared to those who grew up in rural areas. This might be due to the exposure of urban adolescents to media and healthcare facilities than the rural ones.

Similarly, in a study conducted in Germany, residential areas of adolescent girls also showed a statistically significant association with the Uptake of the HPV vaccine [27].

In the current study, those who ever had sexual intercourse were 3 times more likely to take the HPV vaccine than those who didn’t engage in sexual activity, and also those who have perceived risk of acquiring HPV were nearly five times more likely to take the vaccine than those who didn’t. Similarly, in a study conducted in the USA sexually active college women were less likely to get the HPV vaccine [32]. This might be due to the low-risk perception which was indicated in the current study as those who feel more at risk had taken the vaccine.

Furthermore, those who have a mobile phone were nearly two times more likely to receive the HPV vaccine than those who don’t have a mobile phone. This might be due to those who have mobile might be exposed to HPV-related information through the internet.

### Strength

The study has addressed one of the sensitive issues in reproductive health among adolescents**.**

### Limitation

The possibility of social desirability bias and recall bias might not be avoided despite the great effort made. Because the HPV vaccine is given as a school-based program the study can’t generalized for out-of-school adolescents. Also, the nature of the cross-sectional study design makes it difficult to establish cause-and-effect relationships.

## Conclusion

In this study, more than three fourth of the study participants had never heard about Human Papilloma Virus and Vaccine. However, the uptake of the HPV vaccine was nearly two-thirds. The most common reasons reported for not taking the vaccine at all were the myth that the “HPV vaccine would make you infertile” and never knowing or hearing about the vaccine. A place where adolescent girls grow up, having a mobile phone, ever hearing about HPV, Knowledge about the HPV vaccine, ever having sexual intercourse, and perceived risk towards HPV had shown statistically significant association with the uptake of HPV.

### Recommendation

The town health office, Education bureau, schools, and mass media need to work on raising the awareness of adolescent girls on HPV and Vaccines as well as correct the misconceptions regarding vaccination. It would be better to provide due attention to adolescent girls who come from rural areas. Besides, regular school-based health education and the use of mobile technology for health information dissemination have to be considered. Finally, it would be better if a large-scale follow-up study were done to assess the coverage and also, the willingness of those who are eligible but not yet enrolled in the vaccination.

## Data Availability

The datasets used and/or analyzed for this study are available from the corresponding author on reasonable request.

## Abbreviations

AOR: Adjusted Odds Ratio
CI: Confidence Interval
CSA: Central Statistical Agency
E.C.: Ethiopian Calendar
HPV: Human Papilloma Virus
PPE: Personal Protective Equipment
SD: Standard Deviation
TTC: Teachers Training College
USA: United State of America
WHO: World Health Organization
